# Phenotypic and Molecular Convergence of 2q23.1 Deletion Syndrome with Other Neurodevelopmental Syndromes Associated with Autism Spectrum Disorder

**DOI:** 10.3390/ijms16047627

**Published:** 2015-04-07

**Authors:** Sureni V. Mullegama, Joseph T. Alaimo, Li Chen, Sarah H. Elsea

**Affiliations:** 1 Department of Molecular and Human Genetics, Baylor College of Medicine, Houston, TX 77030, USA; E-Mails: mullegam@bcm.edu (S.V.M.); alaimo@bcm.edu (J.T.A.); li.chen@bcm.edu (L.C.); 2 Department of Cellular and Genetic Medicine, School of Basic Medical Sciences, Fudan University, Shanghai 200032, China

**Keywords:** *MBD5*, ASD, networks, overlapping phenotypes, *UBE3A*, *TCF4*, *MEF2C*, *EHMT1*, *RAI1*, transcriptional regulation, pathways, network analysis

## Abstract

Roughly 20% of autism spectrum disorders (ASD) are syndromic with a well-established genetic cause. Studying the genes involved can provide insight into the molecular and cellular mechanisms of ASD. 2q23.1 deletion syndrome (causative gene, *MBD5*) is a recently identified genetic neurodevelopmental disorder associated with ASD. Mutations in *MBD5* have been found in ASD cohorts. In this study, we provide a phenotypic update on the prevalent features of 2q23.1 deletion syndrome, which include severe intellectual disability, seizures, significant speech impairment, sleep disturbance, and autistic-like behavioral problems. Next, we examined the phenotypic, molecular, and network/pathway relationships between nine neurodevelopmental disorders associated with ASD: 2q23.1 deletion Rett, Angelman, Pitt-Hopkins, 2q23.1 duplication, 5q14.3 deletion, Kleefstra, Kabuki make-up, and Smith-Magenis syndromes. We show phenotypic overlaps consisting of intellectual disability, speech delay, seizures, sleep disturbance, hypotonia, and autistic-like behaviors. Molecularly, MBD5 possibly regulates the expression of *UBE3A*, *TCF4*, *MEF2C*, *EHMT1* and *RAI1*. Network analysis reveals that there could be indirect protein interactions, further implicating function for these genes in common pathways. Further, we show that when *MBD5* and *RAI1* are haploinsufficient, they perturb several common pathways that are linked to neuronal and behavioral development. These findings support further investigations into the molecular and pathway relationships among genes linked to neurodevelopmental disorders and ASD, which will hopefully lead to common points of regulation that may be targeted toward therapeutic intervention.

## 1. Introduction

Autism spectrum disorder (ASD) is a growing public health concern that affects millions of individuals worldwide [1]. ASD is a broad term encompassing a heterogeneous group of complex, highly heritable neurodevelopmental disorders in which individuals have impairments in social interaction and communication coupled with repetitive and restricted behaviors [2]. Other morbidities such as intellectual disability, epilepsy, neurological disabilities (ataxia and hypotonia), and other behavioral disorders have been associated with ASD [3]. The molecular etiology of ASD involves the interplay of many genes [4]. A contributor to the risk of ASD resides in high-impact rare variants, such as chromosomal abnormalities, copy number variation (CNV), and mutations in genes previously linked to classic monogenenic genetic disorders such as fragile X syndrome (FXS) (MIM 300624), Rett syndrome (RTT) (MIM 312750), Angelman syndrome (AS) (MIM 105830), and Smith-Magenis syndrome (SMS) (MIM 182290) [2,5,6].

With the advancement of genetic diagnostic technologies such as chromosomal microarray analysis, the identification of novel genetic subtypes of ASD and their associated genes have come to light, such as 16p11.2 deletion (MIM 611913, gene unknown), 2q23.1 deletion syndrome (MIM 156200, *MBD5*), Pitt-Hopkins syndrome (PTHS MIM 610954, *TCF4*), 5q14.3 deletion syndrome (MIM 613443, *MEF2C*), and Kleefstra syndrome (MIM 610253, *EHMT1*). Many of these genes are involved in neuronal functions (synaptic transmission and cell-cell interaction), chromatin modification, methylation, and transcriptional regulation [7]. The identification of these disease genes associated with ASD has led to many theories regarding the pathogenesis of ASD. One theory that has garnered support from some in the ASD community proposes that the pathogenesis is due to the disruption of neurodevelopment, which is triggered by genes with global effects on expression (chromatin modification, methylation, and transcriptional regulation) of other genes that are specifically involved in neuronal functions [7]. Further, these genes are thought to be most likely involved in common ASD-associated pathways such as cell adhesion, cadherin signaling, WNT signaling, PTEN signaling, mTOR signaling, PI3K-Akt signaling, and circadian rhythm [4,8].

Corroborating studies have suggested that a disease phenotype is rarely a consequence of an abnormality in a single gene product but a reflection of dysfunction in a variety of genes and pathobiological processes that interact in complex networks and pathways [9,10,11]. Many neurodevelopmental genetic disorders associated with ASD share common phenotypic features despite their genetic heterogeneity, which further suggests that these genes may act together in complex interconnected pathways that, when perturbed, manifest similar phenotypes.

2q23.1 deletion syndrome (MIM 156200), previously known as “pseudo-Angelman syndrome,” was initially identified in one of the first comparative genomic hybridization (CGH) surveys of developmental disorders [12]. Patients are characterized by severe intellectual disability, seizures, significant speech impairment, and autistic-like behavioral problems [13]. 2q23.1 deletion syndrome is caused by deletion in the chromosomal region 2q23.1 or gene specific deletions in methy-CpG-binding domain 5 (MBD5, MIM 611472). Deletions of 2q23.1 range from small deletions of 38 kb to >19 Mb [13]. While some large deletions extend in the chromosomal region 2q22.3, these deletions do not include the zinc finger E box-binding homeobox 2 gene (*ZEB2*, MIM 605802), the causative gene for Mowat-Wilson syndrome (MIM 235730)*. MBD5* is part of the methyl-CpG-binding domain (MBD) family, which consists of a well-known ASD-associated gene, *MECP2*, which is the causative gene in Rett syndrome. MBD5 has two known isoforms [14] and is thought to have a role in epigenetic modification [7,14,15,16,17,18]. Further, MBD5/Mbd5 has been shown to regulate expression of genes, suggesting it acts as a transcription factor [15,17,19].

In this study, we conducted a comprehensive phenotypic overview of 2q23.1 deletion syndrome and then briefly examined the phenotypic and molecular relationships between 2q23.1 deletion syndrome and neurodevelopmental disorders associated with ASDs. Finally, we examined the pathways in common between 2q23.1 deletion syndrome and Smith-Magenis syndrome.

## 2. Results and Discussion

### 2.1. 2q23.1 Deletion Syndrome Clinical Review

**Figure 1:**
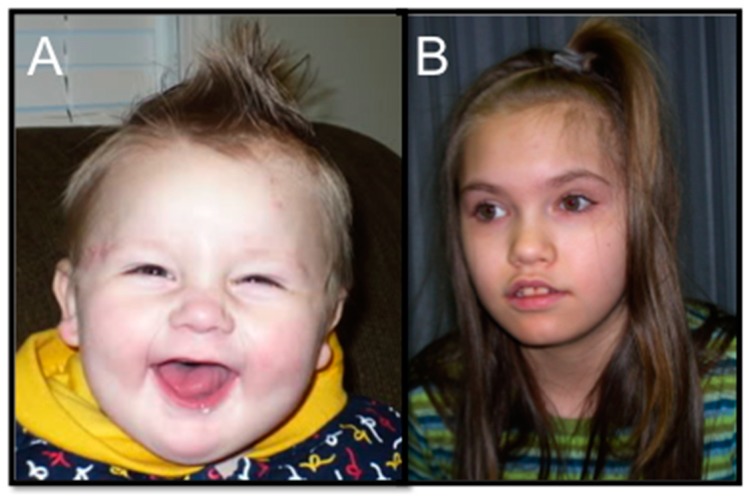
Craniofacial features of 2q23.1 deletion syndrome. Children with 2q23.1 deletion syndrome exhibit broad forehead, open mouth, and tented, thin upper lip. (**A**) 1 year old male. (**B**) 13 year old female. Approvals from parents were obtained to publish these photos.

To further update our phenotypic knowledge of 2q23.1 deletion syndrome, we surveyed the most prevalent phenotypic features of all 2q23.1 deletion cases (*MBD5*-specific deletions and 2q23.1 deletions) using the two largest phenotypic studies on 2q23.1 deletion syndrome [13,20] and case reports that were published subsequently after these studies [15,16,19,21,22,23,24]. Overall, phenotypic findings are comparable across the 74 cases reported [13,15,16,20,21,22,23,25,26,27,28,29,30], and the frequently reported neurological, neurobehavioral, and craniofacial features associated within syndrome are seen and listed in Figure 1 and Table 1 [13,20]. Neurological and behavioral features are the most prevalent findings in 2q23.1 deletion syndrome patients, while the other abnormalities, such as craniofacial abnormalities and skeletal abnormalities, are not consistently the same among individuals with this deletion (Figure 1).

Developmental delay and motor delay were present in 100% of the 2q23.1 deletion cases studied. Previous reports have identified seizures and severe language impairment as two primary features of 2q23.1 deletion syndrome, and we further show that 94.4% and 84.9% of the 2q23.1 deletion cases exhibit these features, respectively [13,15,16,20,21,22,23,25,26,27,28,29,30]. Infantile hypotonia and feeding difficulties were also present in greater than 85% of reported 2q23.1 deletion cases (Table 1). Autistic-like behaviors and behavioral problems were reported in 98.4%. The most prevalent behaviors were distractibility/short attention span (100%) and sleep disturbances (78.8%). These studies also showed that there is a clear lack of precise documentation by clinicians regarding the exact autistic-like behaviors of individuals with 2q23.1 deletion syndrome. Nonetheless, impairments in communication, social interaction and repetitive behaviors are key behavioral criteria for ASD, which are also present in the majority of 2q23.1 deletion syndrome patients. Overall, these patients should be assessed by ADI-R and ADOS to identify specific ASD phenotypes present in individuals with 2q23.1 deletion syndrome. It is apparent that while craniofacial abnormalities are present in children with 2q23.1 deletion, the features are variable across the population. While mild dysmorphic craniofacial features were present at >70% in reported cases, including broad forehead, arched/thick eyebrows, eye abnormalities, nasal abnormalities, downturned corners of the mouth, open mouth, thin upper lip, tented upper lip, and thick or everted lower lip, a consistent and thorough evaluation, measurement, and documentation of craniofacial and skeletal abnormalities is necessary for an accurate and full understanding of the commonly observed associations with 2q23.1 deletion syndrome (Figure 1).

**Table 1:** Prevalent features of 2q23.1 deletion syndrome.

2q23.1 Deletion ^1^		
Common Features	Frequency	Percentage (%)
**Neurological**		
Developmental delay	74/74	100
Motor delay	45/45	100
Language impairment	51/54	94.4
Ataxia	22/32	68.7
Infantile hypotonia	28/30	93.3
Infantile feeding difficulties	17/20	85.0
Seizures	45/53	84.9
**Behavioral**		
Autistic-like behaviors	60/61	98.4
Behavioral problems	60/61	98.4
Aggression/temper tantrums	13/21	62.9
Distractibility/short attention span	21/21	100
Hyperphagia	8/16	50.0
Self-injurious behaviors	21/33	63.6
Sleep disturbances	41/52	78.8
**Growth/Endocrine Abnormalities**		
Postnatal growth retardation	25/51	49.0
Obesity	6/17	35.3
Short stature (<5th percentile)	30/43	69.8
**Craniofacial Abnormalities**		
**Cranium**		
Brachycephaly	12/36	33.3
Broad forehead	21/30	70.0
Microcephaly	28/46	60.9
**Eyes**		
Arched/thick eyebrows	19/24	79.2
Myopia/hypermetropia/corrective lenses	8/11	72.7
Synophrys	13/28	46.4
**Nose/Ear**		
Nasal abnormalities	42/43	97.7
Outer ear abnormalities	22/29	75.9
**Mouth/Chin**		
Dental abnormalities	18/35	51.4
Downturned corners of the mouth	20/28	71.4
Macroglossia or protruding tongue	8/33	24.2
Micrognathia/retrognathia	16/30	53.3
Open mouth	26/34	76.5
Tented upper lip	19/30	63.3
Thin upper lip	21/28	75.0
Thick or everted lower lip	19/26	73.1
Wide mouth	15/25	60.0
**Skeletal Extremity Abnormalities**		
Brachydactyly	14/33	42.4
Clinodactyly, 5th finger	24/38	63.2
Sandal gap	12/32	37.5
Short fifth digit	16/37	43.2
Small hands and feet	25/37	67.6

^1^ Cases came from [13,15,20,21,22,23,24].

### 2.2. Overlapping Phenotypes across 2q23.1 Deletion Syndrome and Other Autism Spectrum Disorders

2q23.1 deletion has been aptly termed a “potent masquerader”, wherein the clinical features of the disorder were initially thought to be due to other well-known genetic syndromes [20]. Since the neurodevelopmental and behavioral characteristics common to 2q23.1 deletion syndrome are nonspecific and commonly found in multiple other neurodevelopmental disorders associated with ASD [20], many 2q23.1 deletion syndrome patients were initially tested for a variety of disorders, including Rett, Angelman, and Smith-Magenis syndromes [26]. As previously mentioned, Mowat-Wilson syndrome and 2q23.1 deletion syndrome are on neighboring chromosome bands, patients with 2q23.1 deletion do not share key anomalies associated with Mowat-Wilson syndrome, such as Hirschsprung disease, congenital heart defects, genitourinary anomalies, and eye defects; thus, Mowat-Wilson syndrome is not in the differential diagnosis for 2q23.1 deletion syndrome patients [31,32]. Here, we reviewed the neurological and behavioral features of 2q23.1 deletion syndrome and eight other disorders that have been phenotypically linked to or considered in the differential diagnosis for 2q23.1 deletion syndrome [31,32]. The reported phenotypic features of 2q23.1 deletion syndrome, RTT, AS, PTHS, 2q23.1 duplication syndrome, 5q14.3 deletion syndrome, Kleefstra syndrome (KFS), Kabuki syndrome (MIM 147920), and SMS show they share neurological and behavioral co-morbidities coupled with ID, including motor impairments and gaiting abnormalities, hypotonia, language impairments, seizures, sleep disturbance, autistic-like behaviors, and other distinctive behaviors (Table 2). The similar features among many highly penetrant neurodevelopmental disorders associated with ASD allow us to hypothesize several models for ASD etiology and pathogenesis. First, the ASD-associated genes linked to known neurodevelopmental disorders govern similar cellular functions. Second, the genes play a role in specific cellular functions that converge into common molecular pathways that are associated with specific phenotypes. Third, these genes are involved in entirely different molecular pathways that converge to a common phenotype. 

### 2.3. MBD5 Regulates Disorder-Specific Genes

**Figure 2:**
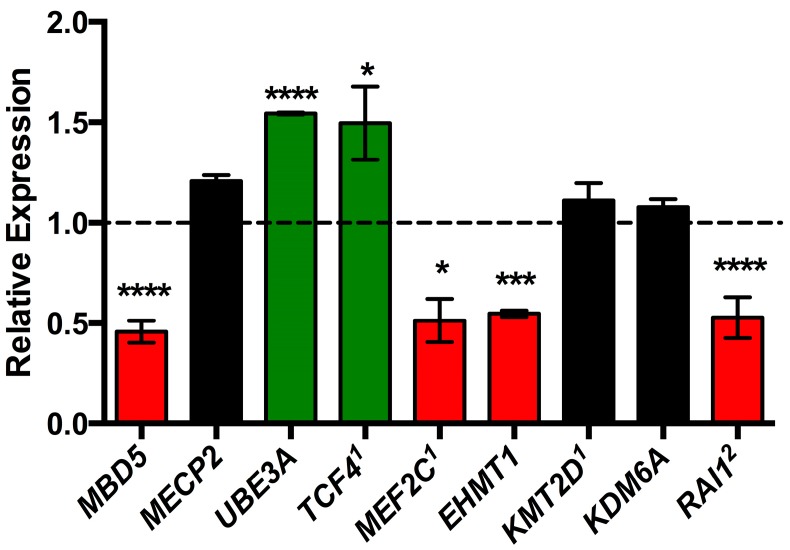
Dysregulation of associated genes in 2q23.1 deletion syndrome. *MBD5*, *MEF2C*, *EHTM1*, and *RAI1* were significantly down regulated (red bars) while *UBE3A* and *TCF4* were significantly up regulated (green bars). *MECP2*, *KMT2D*, and *KDM6A* did not have altered mRNA levels (black bars). Gene expression is shown relative to control set to 1.0 (black line). Graphs represent mean ± SEM (* *p* < 0.05, *** *p* < 0.01, **** *p* < 0.0001). 1 = Manuscript in review, 2 = [20].

Due to the high degree of phenotypic similarity between RTT (*MECP2*), AS (*UBE3A*), PTHS (*TCF4*), 2q23.1 duplication syndrome (*MBD5*), 5q14.3 deletion syndrome (*MEF2C*), KFS (*EHMT1*), Kabuki syndrome (*KMT2D* and *KDM6A*), and SMS (*RAI1*) relative to 2q23.1 deletion syndrome (Table 2), we examined the co-expression relationships of these genes to MBD5 toward assessing their involvement in the phenotype of 2q23.1 deletion syndrome. We hypothesized that these genes may be dysregulated in 2q23.1 deletion syndrome and therefore assessed expression of *MECP2*, *UBE3A*, *TCF4*, *MEF2C*, *EHMT1*, *KMT2D*, *KDM6A*, and *RAI1* in 2q23.1 deletion syndrome patient lymphoblastoid cell lines (LCLs) that had various deletions of *MBD5*. As expected, mRNA levels of *MBD5* were significantly down regulated in 2q23.1 deletion syndrome LCLs (*p* < 0.0001), confirming previously reported results [13] (Figure 2). Overall, we observed 5/8 (62.5%) of the tested genes had significantly altered mRNA levels when *MBD5* was haploinsufficient. *TCF4* and *UBE3A* expression levels were elevated to ~1.5-fold (*TCF4*, *p* = 0.012; *UBE3A*, *p* < 0.0001). However, *MEF2C*, *EHMT1*, and *RAI1* expression levels were significantly reduced to ~0.5-fold (*MEF2C*, *p* = 0.016; *EHMT1*, *p* = 0.0004; *RAI1*, *p* < 0.0001). *MECP2*, *KMT2D*, and *KDM6A* had no significant change of expression.

Our co-expression data indicate that *MBD5* functions, either directly or indirectly, in the regulation of the expression of other ASD-associated genes (*TCF4*, *MEF2C*, *EHTM1*, *RAI1*, and *UBE3A*), which supports the common pathway ASD pathogenesis hypothesis. *MBD5* and the genes with altered expression possibly converge on common pathways that contribute to the phenotype of 2q23.1 deletion syndrome and genetic neurodevelopmental syndromes associated with ASD. While *MECP2*, *KMT2D*, and *KDM6A*, did not have altered mRNA levels, these genes may share a molecular connection to *MBD5* beyond co-expression, such as physical interactions. Thus, MBD5 and *MECP2*, *KMT2D*, and *KDM6A* could be involved in different genetic pathways that result in a similar phenotypic output, which may indicate the presence of redundant pathways. Alternatively, *MECP2*, *KMT2D*, and *KDM6A* may be in the same or overlapping pathways but may function upstream of MBD5. Our phenotypic and gene expression data (Table 2 and Figure 2) implicate some form of pathway overlap or interaction when *MBD5* is haploinsufficient.

**Table 2:** Common phenotypes between 2q23.1 deletion syndrome and Rett, Angelman, Pitt-Hopkins, 2q23.1 duplication, 5q14.3 deletion, Kleefstra, Kabuki, and Smith-Magenis syndromes.

DISORDER	2q23.1 del	RTT	AS	PTHS	2q23.1 dup	5q14.3 del	KFS	KMS	SMS
**Key References**	[13,20]	[33,34]	[31,35]	[36,37]	[38,39]	[40]	[41]	[42,43]	[44]
**Causative Gene**	*MBD5*	*MECP2*	*UBE3A*	*TCF4*	*MBD5*	*MEF2C*	*EHMT1*	*KMT2D*, *KDM6A*	*RAI1*
**Neurological/Behavioral Characteristics**									
Intellectual disability ^a^	+++	+++	+++	+++	++	+++	+++	++	++
Speech delay ^b^	+++	+++	+++	+++	++	+++	++	++	+
Seizures ^c^	+++	+++	+++	++	++	++	++	+	+
Sleep disturbance ^d^	+++	+	+++	++	++	+	+	+	+++
Delayed walking ^e^	++	+++	++	++	+	+++	+	+	+
Hypotonia	+	+	+	+	+	+	+	+	+
Autism-like behaviors	+	+	+	+	+	+	+^f^	+	+
Feeding difficulties	+	+	+	-	+	+	+	+	+
Stereotypic behaviors	+	+	+	+	+	+	-	+	+
Ataxia	+	+	+	+	+	+	-	+	-
Happy disposition (frequent or inappropriate laughing)	+	+	+	+	+	NR	-	+	-
Hyperactivity/short attention span	+	-	+	+	+	-	-	+	+
Self-injurious behavior	+	-	-	-	-	-	+	-	+
Aggressive behavior	-	-	-	+	-	-	+	-	+

2q23.1 del = 2q23.1 deletion syndrome, RTT = Rett syndrome, AS = Angelman syndrome, PTHS = Pitt-Hopkins syndrome, 2q23.1 dup = 2q23.1 duplication syndrome, 5q14.3 del = 5q14.3 deletion syndrome, KFS = Kleefstra syndrome, KMS = Kabuki make-up syndrome, and Smith-Magenis syndrome, ^a^ + = mild; ++ = moderate; +++ = severe, ^b^ + = moderate; ++ = severe; +++ = absent, ^c^ + = 0%–40%; ++ = 41%–70%; +++ 71%–100%, ^d^ + = 0%–40%; ++ 40%–70%; +++ 71%–100%, ^e^ + = 1−3 y; ++ = 4−6 y; +++ = >6 y or limited mobility, ^f^ only in childhood, NR = not reported.

### 2.4. MBD5 Network

**Figure 3:**
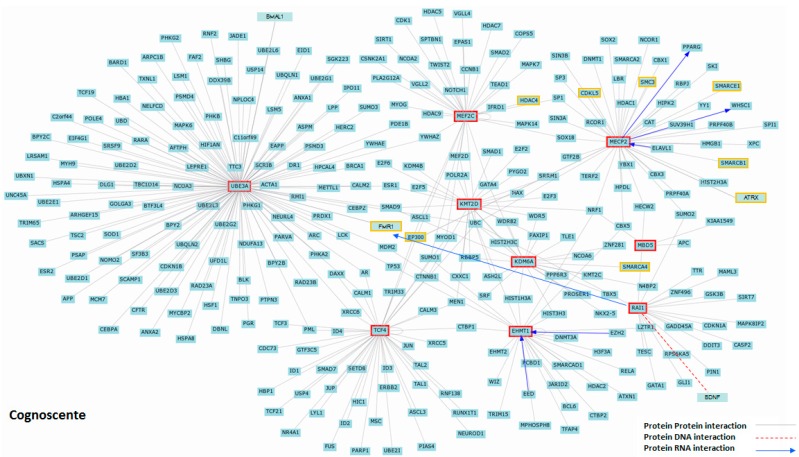
2q23.1 deletion syndrome gene interaction network. Cognoscente was used to generate direct protein-protein (grey line), protein-DNA (red dash line), and protein-RNA (blue arrow line) interactions of genes previously implicated in ASD: *MBD5*, *MECP2*, *UBE3A*, *TCF4*, *MEF2C*, *EHMT1*, *KMT2D*, *KDM6A* and *RAI1* (red box). The human orthologs of genes of many of the protein products that came up in this network are involved in neuronal functions, chromatin modification, methylation and transcriptional regulation. In addition, other genes associated with ASD also were identified (yellow box).

Recent studies suggest that genes involved in common endpoints (e.g., phenotypes) show an increased tendency to have protein-protein interactions, similar expression patterns in specific tissues, and exhibit synchronized expression as a group [45,46]. In this study, we observed co-expression relationships between *MBD5* and ASD-associated genes (Figure 2). To comprehensively understand MBD5-dependent genetic pathways relative to these genes, we utilized bioinformatics resources and the network tool, Cognoscente (http://vanburenlab.tamhsc.edu/), to uncover related protein interactions (Figure 3). While data do not exist in the literature to support the key genes (in red boxes) directly interacting with each other, we did observe that some hubs (central network connections) all intervened with one another (Figure 3). This finding could suggest there are intermediary genes that molecularly connect the genes directly involved in known disorders. Interestingly, from this network, several other genes linked to ASD were identified, including *CDKL5*, *HDAC4*, *EP300*, *FMR1*, *SMARCA4*, and *ATRX*, each previously linked to a neurodevelopmental disorder with overlapping or similar phenotypes, as listed in Table 2 (Figure 2). In Mullegama *et al.* (2014), we observed overexpression of *FMR1* in 2q23.1 deletion syndrome cell lines [19]. Additional expression studies of these ASD-associated genes in 2q23.1 deletion syndrome would prove interesting. A limitation using such tools to explore molecular networks, as we demonstrated in Figure 2, is that all programs that generate networks, like Cognoscente, rely on the published literature [47]. This fact revealed that there is clearly a lack of research and understanding of the interactions among the genome and proteome in ASD. Thus, undoubtedly, concerted efforts need to be made to study molecular interactions between these genes collectively to further elucidate the genetic etiology of these and other monogenic neurodevelopmental disorders associated with ASDs. Futures studies such as RNA-seq, methylation sequencing, whole-genome miRNA analysis, and chromatin immunoprecipitation sequencing (ChIP-seq) studies of these disorder-causing genes will allow us to further elucidate the levels of molecular convergence between these disorders.

### 2.5. Molecular Relationships between MBD5 and RAI1

We propose that functional relationships between genes serve as a strong indicator for involvement in key pathways responsible for phenotypic outcomes. An example of this concept is the relationship between MBD5 and RAI1*.* We have shown that 2q23.1 deletion syndrome and Smith-Magenis syndrome have overlapping phenotypes (see Table 2). From the data we presented here, it appears that *RAI1* could be regulated directly or indirectly by MBD5*.* Our previously published work also suggested that *RAI1* is dysregulated when *MBD5* is knocked down through siRNA technology in neuroblastoma SH-SY5Y cell lines [19]. Therefore, we wanted to further examine the functional pathways common to *MBD5* and *RAI1*.

From previously published microarray data [19] where *MBD5* and *RAI1* were knocked down through siRNA technology individually in neuroblastoma cell lines, we conducted Ingenuity Pathway Analysis (IPA) on these microarray data to identify the common pathways that are perturbed when *MBD5* and *RAI1* are haploinsufficient. We propose that these pathways could be key contributors to the overlapping phenotypes we see in both syndromes. IPA was used to determine which biological pathways and function involving genes were differentially expressed in *MBD5* and *RAI1* knockdown SH-SY5Y cell lines. A *p*-value of less than 0.05 for each pathway was determined using Fisher’s exact tests to determine the likelihood of those genes assigned by chance. IPA shows that *MBD5* and *RAI1* are implicated in many neurological, cell growth and developmental pathways (*p* < 0.05) (Table 3 and Table 4). The top common pathways reveal many interesting and important pathways altered due to *MBD5* and *RAI1* haploinsufficiency. Previously mentioned pathways that are associated with autism and sleep, circadian rhythm signaling, and mTOR signaling pathways are present (Table 3 and Table 4) [19]. The CDK5 signaling pathway is crucial to neuronal activity, neuronal migration during development and neurite growth [48]. In previous mouse studies of Mbd5, reduced neurite outgrowth was observed [49]. Further, the *Xenopus laevis* rai1 morphants exhibit aberrant neural crest migration [50]. Thus, the CDK5 signaling pathway could be contributing to the features seen in the above studies. The apoptosis signaling pathway (see Table 4) is involved in the determination of the size and shape of the brain and it regulates the wiring of developing neuronal networks [51]. It is thought that dysregulation of this pathway can lead to neuroanatomic abnormalities and developmental disabilities [51]. There are growing associations between neural cell death and autism [51]. Thus, the involvement of the apoptosis signaling pathway in 2q23.1 deletion syndrome and Smith-Magenis syndrome should be further investigated. Overall, extensive molecular studies on how these shared pathways contribute to the phenotypes present in 2q23.1 deletion syndrome and Smith-Magenis syndrome is necessary. In addition, these results provide further evidence that monogenic neurodevelopmental disorders associated with ASD share common pathways and are crucial to study in regards to phenotypic development.

**Table 3:** Common neurological pathways in both *MBD5* and *RAI1* knockdown SH-SY5Y cells.

Ingenuity Canonical Pathways *
Acetate conversion to acetyl-CoA
Agrin interactions at neuromuscular junction
Aldosterone signaling in epithelial cells
Amyotrophic lateral sclerosis signaling
Axonal guidance signaling
Cyclin-dependent kinsase 5 signaling
Choline degradation I
Circadian rhythm signaling
Ciliary neurotrophic factor signaling
Docosahexaenoic acid signaling
Dolichol and dolichyl phosphate biosynthesis
Eukaryotic initiation factor 2 signaling
Ephrin A signaling
Ephrin B signaling
Ephrin receptor signaling
Epidermal growth factor receptor signaling
G protein signaling mediated by Tubby
Gap junction signaling
Glial cell line-derived neurotrophic factors family ligand-receptor interactions
Glioma signaling
Melanoma signaling
Mechanistic target of rapamycin signaling
Netrin signaling
Neuregulin signaling

* All pathways had a *p*-value <0.05 as determined by Fisher Exact Test within Ingenuity Pathway Analysis.

**Table 4:** Common cell growth and developmental pathways in both *MBD5* and *RAI1* knockdown SH-SY5Y cells.

Ingenuity Canonical Pathways *
14-3-3-mediated signaling
1D-myo-inositol hexakisphosphate biosynthesis II (mammalian)
Acetate conversion to acetyl-CoA
Agrin interactions at neuromuscular junction
Aldosterone signaling in epithelial cells
Angiopoietin signaling
Apoptosis signaling
Aryl hydrocarbon receptor signaling
Assembly of RNA polymerase II complex
Assembly of RNA polymerase III complex
Ataxia telangiectasia mutated signaling
Bone morphogenetic protein signaling
CD40 signaling
Cell division control protein 42 homolog signaling
Cytidine diphosphate diacylglycerol-diacylglycerol biosynthesis I
Cell cycle control of chromosomal replication
Cell Cycle: G1/S checkpoint regulation
Cell Cycle: G2/M DNA damage checkpoint regulation
Ceramide signaling
Cholecystokinin/gastrin-mediated signaling
Choline degradation I
Circadian rhythm signaling
Citrulline-nitric oxide cycle
Clathrin-mediated endocytosis signaling

* All pathways had a *p*-value <0.05 as determined by Fisher Exact Test within Ingenuity Pathway Analysis.

## 3. Experimental Section

### 3.1. Patients and Cell Culture Studies

All samples and information were collected after informed consent was obtained and in accordance with Institutional Review Board (IRB) approved protocols from Baylor College of Medicine. Lymphoblastoid cell lines (LCLs) (Epstein–Barr virus-transformed human lymphocytes) were cultured as previously described [25]. LCLs utilized in this study included: 2q23.1 deletion syndrome (SMS367 [25], SMS185 [25], SMS361 [13], SMS373 [13], SMS375 [13], and SMS368 [13] and five normal control lines).

### 3.2. Expression Analyses

RNA was isolated from cultured patient and control lymphoblastoid cell lines via TRIzol (Invitrogen, Carlsbad, CA, USA) according to standard protocols. All cell lines were cultured for the same period of time to the same cell density. RNA was quantified using the NanoDrop^®^ ND-100 Spectrophotometer (NanoDrop Technologies, Inc., Wilmington, DE, USA). First-strand cDNA synthesis was carried out using qSCRIPT cDNA SuperMix (Quanta Biosciences, Inc., Gaithersburg, MD, USA) (with 1 µg of RNA) according to the manufacturer’s protocol. For quantitative real-time PCR, predesigned Taqman MGB probes from Assays-on-Demand Gene Expression Products (ABI) (Life Technologies Inc., Carlsbad, CA, USA) were used for all genes. A minimum of three unrelated patient samples were run in triplicate in 10 µL reaction volumes. PCR conditions were the default settings of the ABI Prism 7900 HT Sequence Detection System (Life Technologies Inc., Carlsbad, CA, USA). The cycle threshold (*C*_t_) was determined during the geometric phase of the PCR amplification plots as recommended by the manufacturer. Relative differences in transcript levels were quantified with the ΔΔ*C*t method with *GAPDH* (MIM138400) (Hs99999905_m1) mRNA as an endogenous control. All expression values were calculated relative to control levels set at 1.0.

### 3.3. Network Analysis

Cognoscente (http://vanburenlab.tamhsc.edu/) was used to identify the biomolecular interactions that have been documented in the literature of the genes listed in Table 2. Cognoscente is a highly curated freely available database that identifies interacting components of protein networks and the primary literature to support such interactions [47].

### 3.4. Ingenuity Pathway Analysis (IPA)

IPA was used to identify biological functions, gene networks and pathways, and likely upstream regulators that were significantly more altered in knockdown cells than in controls. Significant interactions were determined using the Ingenuity Pathway Knowledge Base and a Fisher’s exact test to calculate a *p*-value determining the probability that each function network or pathway assigned to that data set is due to chance alone. Statistical significance was determined at a cutoff of *p* ≤ 0.05.

### 3.5. Statistical Analyses

Statistical analysis for gene expression data was performed with Prism 4 version 4.0b (GraphPad Software, Inc., San Diego, CA, USA). Statistical significance was determined at a cutoff of *p* ≤ 0.05.

## 4. Conclusions

In conclusion, we show that 2q23.1 deletion syndrome shares common neurological and behavioral phenotypes with other monogenic neurodevelopmental disorders associated with autism spectrum disorders. Furthermore, haploinsufficiency of *MBD5* impacts expression of several ASD-implicated genes, including *UBE3A*, *TCF4 MEF2C*, *EHMT1*, and *RAI1*; supporting MBD5 as a transcriptional regulator. Further, data suggest that these genes associated with ASD, *MBD5*, *MECP2*, *UBE3A*, *TCF4 MEF2C*, *EHMT1*, *KMT2D* and *KDM6A*, and *RAI1* may be part of a gene network that converges into common or overlapping pathways that results in similar phenotypes when perturbed. As an example, we show that when *MBD5* and *RAI1* are both haploinsufficient they share common perturbed pathways that likely contribute to the overlapping phenotypes exhibited by 2q23.1 deletion syndrome and Smith-Magenis syndrome patients. Overall, future studies identifying and dissecting the genetic and downstream molecular pathways in monogenic neurodevelopmental disorders associated with ASD may reveal common points of regulation and promote gene candidates for targeted therapeutic and pharmacological intervention.
